# Glass Ionomer Cements for the Restoration of Non-Carious Cervical Lesions in the Geriatric Patient

**DOI:** 10.3390/jfb9030042

**Published:** 2018-07-08

**Authors:** Nikolaos-Stefanos Kampanas, Maria Antoniadou

**Affiliations:** Department of Operative Dentistry, School of Dentistry, National and Kapodistrian University of Athens, 115 27 Athens, Greece; mantonia@dent.uoa.gr

**Keywords:** glass ionomer cements, geriatric dentistry, elderly patients, non-carious cervical lesions

## Abstract

Background: The restoration of non-carious cervical lesions in geriatric patients is a demanding process. Glass ionomer cements can be promising materials for the management of these lesions in older adults. The aim of this literature review is to present the benefits of glass ionomers and how they can be used for the restoration of non-carious cervical lesions of older adults depending on the geriatric patient’s profile. Data sources: All available in vitro and in vivo studies from Google Scholar, PubMed and Scopus search engines corresponding to glass ionomer cements, geriatric dentistry, elderly patients, and non-carious lesions as key words were reviewed. Data synthesis: The advantages of glass ionomer cements, such as good retention and fluoride release, make them suitable for the restoration of non-carious cervical lesions. However, several factors related to the geriatric patient’s profile determine the most suitable material type. Conclusion: In general, the resin modified glass ionomer cements (RMGICs) appear to be preferred, but under certain circumstances the use of the conventional product is more appropriate, despite its poorer mechanical features. Further studies are required for more reliable data analysis and clinical interpretation of the relevant results.

## 1. Introduction

The restorative management of non-carious cervical lesions (NCCLs) presents a special challenge due to their histological and structural features. Although the creation of these lesions is complexed, the main etiological factors are erosion, abrasion and abfraction. Erosion describes loss of hard dental tissue due to chemical acids, such as vinegar beverages, and abrasion due to mechanical factors, such as tooth brushing. The abfraction theory is used to describe the breakdown of hard dental tissue in the cervical area caused by the accumulation of tensile forces during heavy non-axial occlusal loading. The margins of NCCLs may be located in enamel, cementum or dentin and often have a subgingival position in an aqueous environment. In some cases, part of the cavity is not reachable or cannot be isolated properly. Although many studies have evaluated the use of resin composites for the restoration of NCCL, glass ionomer cements are also promising materials for the management of these lesions [1,2].

Nowadays, the number of people regarded as older adults or as elderly is rapidly increasing [3]. Due to preventative dentistry and higher life-span, the maintenance of teeth of the elderly has seen significant growth [4]. This fact, along with changes in the human diet, has multiplied the prevalence of non-carious cervical lesions in older people [5]. Oral and more general health problems combined with the mental and communication limitations of these patients create further difficulties in restoring NCCL [6]. Research regarding restoration of these lesions with the use of glass ionomer cement in elderly patients is limited. Thus, the aim of this study is to present the advantages of glass ionomers and how they can be used successfully for the restoration of non-carious cervical lesions of older adults depending on the geriatric patient’s profile.

## 2. Materials and Methods

A literature search was conducted using the online databases PubMed, Google scholar and Scopus, searching for original research papers and review articles concerning glass ionomer cements, non-carious cervical lesions and geriatric patients. The electronic search covered the period from January 2002 to October 2017. The combination of the following terms was used to identify relevant publications: “glass ionomer cements” OR “geriatric dentistry” OR “elderly patients” OR “non-carious cervical lesions”. The criteria for the final inclusion of each article after full text retrieval were: review articles, in vitro and clinical studies, case series or case reports, selecting those which also cover the restoration of non-carious cervical lesions with glass ionomer cements or the needs of the geriatric patients, suitable information with regard to the materials used, outcome based on clear data from laboratory and clinical examination, and articles written in the English language. Exclusion criteria were: Articles irrelevant to glass ionomers, non-carious cervical lesions and geriatric patients, animal studies, inadequate data regarding the application of the material used, articles written in languages other than English, conference proceedings, and inaccessible abstracts. The criteria were applied to each article independently by the reviewers for reliable results. The final sample was formed of 28 articles. A limitation in the literature was the absence of articles studying the application of glass ionomers exclusively for the restoration of non-carious cervical lesions in geriatric patients.

## 3. Glass Ionomer Cement: Retention in NCCL and Longevity in the Oral Environment

There is a satisfying number of clinical studies of retention rates of resin modified glass ionomer (RMGI) used to restore NCCL over one to thirteen years, showing retention rates of 85.7% to 100% [7,8,9]. In another recent study, Class V restorations placed by UK general practitioners over a two-year period were evaluated. Between the materials, resin modified glass ionomer recorded the smallest percentage of early failure. Retention of RMGI was reported at 91.4%, when composite resins showed 85.3% in the same study [10]. In general, retention rates for resin composite restorations are lower in comparison with resin modified glass ionomer restorations [10]. A clinical trial was designed to evaluate the two-year clinical performance of a one-bottle etch-and-rinse bonding system associated with a hybrid resin composite compared with a resin modified glass ionomer cement (RMGIC) in NCCL. The researchers observed that the RMGIC product showed a superior retention rate of 100%, with the resin composite system showed a rate of only 78.8% [1]. Retention of conventional glass ionomer cement (GIC) after ten years has been reported at 83% for similarly restored lesions [7,8]. Glass ionomer restorations have survived adequately well in the root surface environment. Restorations showed a collective survival rate of about 77% at 80 months [11].

Resin modified glass ionomers are capable of bonding chemically to the tooth structure. Furthermore, the elastic modulus of RMGIs can offset the fatigue stress caused by tensile forces that are transferred to the cervical part of the tooth due to mastication and malocclusion. The combination of these properties in addition with a coefficient of thermal expansion similar to that of tooth structures lead to better permanency of restorations, especially in enamel margins which are common in non-carious cervical lesions. Therefore, glass ionomer continues to be the most retentive material for the restoration of NCCL [1,5].

## 4. Glass Ionomer Cement: Fluoride Release and Secondary Caries Prevention

Whenever a restoration takes place, there is the possibility for a microspace to be created between the restorative material and the tooth substrate. This microgap in combination with several other factors, such as the regulatory capacity of saliva, caries experience, dietary habits, access to dental care, level of personal oral hygiene, dental knowledge, periodontal disease, gingival recession and general medical conditions, could lead to the appearance of secondary caries around a restoration. In order for the material to resist secondary caries development it should have the ability to release caries-protective agents, such as fluoride or antimicrobials [8,12]. Therefore, one of the properties of glass ionomer cement that made them popular in the dental community is their ability to release fluoride to the restored tooth and the oral environment [7,13].

Glass ionomer cement as a fluoride-releasing dental material provides improved resistance against primary and secondary caries in coronal and root surfaces by several different mechanisms. The release of fluoride into the local environment may slow demineralization. Just 0.08 ppm of fluoride is capable of reversing the process of demineralization of sound tooth structure. Fluoride released from restorative materials is absorbed by the hydroxyapatite crystal surface resulting in the creation of a more acid resistant fluoroapatite veneer. Fluoride, in very low levels, can also take part in the remineralization of lesions and hypomineralized tooth structure. In addition, saliva and plaque absorb fluoride, which affects the bacteria in dental plaque by decreasing the adherence of bacteria to hydroxyapatite and dysregulating their enzyme systems, which are necessary for glycolysis and energy production. Once inside the bacteria, the hydrogen fluoride acidifies the bacterial cytoplasm and leads to the release of fluoride ions. These ions interfere with enzymes essential for the bacterial metabolism (enolase, acid phosphatase, pyrophosphatase, pyrophosphorylase, peroxidase, catalase, proton-extruding ATPase). Several clinical studies have shown reduction of up to 77 percent in cariogenic bacteria within plaque adjacent to glass ionomers, even up to six months after restoration placement [12]. Laboratory findings show that a fluoride concentration of 2 mg/L inhibits the formation of carious lesions by inhibiting bacterial plaque formation and encouraging the formation of fluoroapatite [14].

In comparison to other fluoride-releasing restorative materials, such as compomer and fluoride-releasing composite resin, conventional glass ionomer takes the first place when it comes to fluoride release. One study supported that glass ionomer (GI) released more fluoride compared to the compomers and giomers under study [15]. Results from another trial, with a long follow-up period, suggest that conventional GIC releases over five times more fluoride than compomer and over 21 times more than fluoride-containing composite resin after 12 months (480+/−42) μg/cm^2^; (87+/−17) μg/cm^2^ and (22+/−2) μg/cm^2^, respectively—ANOVA *p* < 0.001—[14].

RMGIs release fluoride to a lesser extent than conventional glass ionomers. Release of fluoride from various RMGIs during the first 24 h reaches a maximum of 5–35 μg/cm^2^ depending on the storage environment. Daily fluoride release begins from 8 ppm to 15 ppm on the 1st day, decreases to 1–2 ppm on the 7th day, and stabilizes in 10 days to 3 weeks [12,15].

Besides the higher release of fluoride, glass ionomers have another crucial advantage. Their unique chemistry allows them to be recharged with fluoride over time. After the placement of a glass ionomer restoration, an ion exchange between saliva and the material takes place, with the fluoride ions diffusing from the GIC to the tooth, following the path to the area of lower fluoride concentration. The unreacted glass ionomer filler can be recharged with fluoride-containing oral care products, including topical fluoride gel applications, fluoride-containing toothpastes and mouth rinses. Substantial increases in fluoride release can be accomplished even with short periods of exposition to only a 50 ppm fluoride solution [7,12].

Many clinical and in vitro studies have assessed the association between fluoride release and remineralization effects on secondary caries prevention in glass ionomers. Specifically, in vitro remineralization of early carious lesions has been examined with Fuji IXGP (GIC), Ketac-Molar (GIC), Vitremer (RMGI), and Z250 (resin composite) used as restorative materials. The extent of caries lesions adjacent to cavities restored with glass ionomers was considerably decreased [11]. Moreover, the percentage of mineral loss from in vitro lesions adjacent to or up to 7 mm from glass ionomer restorations was reduced significantly. The use of glass ionomer reduced mineral loss by almost 80 percent at 0.2 mm from the restoration to 37 percent at 7 mm from the restoration [12]. In a clinical study of 16 restorations applied to Class V non-carious lesions, no secondary caries were detected after five years. This observation was similar to another report (2001) in which no recurrence of caries was found after three years in 82 restorations of a similar type [8].

In order to enhance the anticariogenic effect of glass ionomers, many in vitro attempts have taken place with the addition of antibacterial agents inside glass ionomers. It is demonstrated that the incorporation of 1% chlorhexidine (CHX) diacetate in glass ionomer cement is optimal for providing antibacterial activities [16]. Glass ionomer cement containing ciprofloxacin, metronidazole and minocycline can more effectively inhibit Streptococcus mutans, while one study detected a decrease in more than 98% of bacteria counts isolated from infected dentine after partial caries removal of deciduous teeth in children and sealing with glass ionomer cement contained 1% metronidazole, 1% ciprofloxacin and 1% cefaclor. An in vitro study also found that incorporation of doxycline hyclate significantly improves the inhibitory action of RMGIC. When applying doxycline in concentrations of 1.5%, 3.0% and 4.5%, only the highest was effective against tested bacteria after seven days. *Lactobacillus acidophilus* was the most sensitive bacteria to the antibiotic [17].

Atraumatic Restorative Treatment (ART) is a minimal intervention approach in which demineralized tooth tissues are removed with manual instruments, and the cavity is restored with a filling material [16]. These modifications with antibacterial agents can be used in the future when conditions do not allow complete removal of caries, however they are presently only at an experimental stage.

## 5. Non-Carious Cervical Lesions: Histological Attention Points

Regarding histological features that should be considered before restoring a NCCL, a study analyzed ten undecalcified anterior human teeth with wedge-shaped NCCLs by using red dye penetration from the pulp chamber and scanning electron microscopy. The researchers found that beneath the intact facial enamel, the dentinal tubules were generally patent. However, dentine visible on the ceiling of the wedge was sclerotic, as was the dentine on the floor. These tracts of sclerotic dentine followed the primary curvature of the tubules beneath of the lesions, and their pulpal orifices were closed with reparative dentine. Different levels of observation with SEM revealed that intratubular dentine formation affected both the number of dentinal tubules per square millimeter and the diameters of tubules within the floor of the lesion. Most tubules were obliterated and sclerotic at deeper levels. These results are highly important to clinical practice. The sclerotic dentin resists demineralization and consequently does not respond well to etching and bonding techniques, which require the formation of a hybrid layer enhanced by the penetration of the bonding agents in microporosities of demineralized dentin [18].

## 6. The Profile of Gerodontic Patients

Depending on their ability to seek dental services, the elderly could be categorized into three broad functional groups. The first group is the *functionally independent* older adults, who live unassisted and can access dental care independently using their own vehicles or public transportation. They may have chronic medical problems, such as hypertension, type II diabetes, osteoarthritis, etc., and they correspond to 70% of the ageing community. The second one is the *frail* older adults, who are partly dependent on the help of friends and family for their access to dental services. They comprise about 20% of the population over the age of 65 years. The last group consists of people who are not able to survive in the community independently and are either home-bound (about 5% of the population over 65) or live in institutions or hospitals (another 5% of the population over 65). These *functionally dependent* older adults can only have access to dental services if they are transported to a dental office or, when this is impossible, the services are brought to them through mobile programs [19]. Portable dental equipment, such as a domiciliary valise or a portable dental office, can be used to take care of patients of this group at their nursing home or hospital [20]. Oral health of the hospitalized elderly is poor and most of them are in need of restorative dental treatment. However, functional disability and autonomy loss of these patients is an obstacle in care implementation and often limits care to emergency cases. Many of them are unable to open their mouth properly or can only keep it open for a few moments. As a result, the duration of dental intervention must be as short as possible [21,22,23]. Under these conditions Atraumatic Restorative Treatment may be the most suitable choice of dental care. GIC is considered to be the material of choice [13,14]. A meta-analysis showed that ART restorations in posterior Class V cavities in permanent teeth have a 28% higher chance of being successful than that of amalgam restorations after 6.3 years [14].

The increase in life-span associated with the application of preventive dentistry concepts has resulted in the maintenance of the teeth of older adults. Nevertheless, geriatric patients suffer a large variety of problems which are associated with increasing age. Changes in the oral mucous membrane, missing dentition and tooth loss, periodontal disease, coronal dental caries and root surface caries, secondary caries at old restorations, oral pre-cancer and cancer are some examples. Diminished function of the salivary gland and reduced unstimulated salivary flow is another important issue leading to dryness of the mouth and xerostomia. All these conditions combined with a chronic disease background, medication and the inability to perform adequate oral hygiene could lead patients to experience severe oral problems, including high levels of dental caries, and difficulties in chewing, eating and communicating [3,4,12,24].

There are limited studies concerning the prevalence of NCCLs in the elderly population. One study in China showed that 56.6% of 393 older adults appear to have these lesions, with 81.3% of teeth being affected. 49.0% of the elderly subjects examined had at least one tooth with NCCL deeper than 1 mm, which needed restoration. If the edentulous subjects were eliminated, these percentages are increased to 86.1% and 51.9%, respectively. The explanation of these high rates may be that teeth have been exposed to the etiological factors of NCCL for a longer time and are sometimes in combination with a higher prevalence of gingival recession and root exposure. It is also remarkable that the most frequently affected tooth was the first premolar, followed by the second premolar and the canine [25]. That could be explained by the fact that the tooth surfaces on which NCCLs develop have been shown to be those least protected by serous saliva from the major salivary glands, a situation much more intense in older patients with decreased flow of saliva or salivary gland disorders [18].

## 7. Discussion

When it comes to the restoration of NCCLs with GIC, the first step that should concern the clinician is the right cavity preparation. The presence of sclerotic dentin still remains an obstacle to obtaining good bonding with RMGIC, and results in the creation of microleakage [5]. The bonding mechanism of RMGI is two-fold, including a chemical interaction with the tooth structure based on the ionic binding of the multiple carboxylic groups of polyalkenoic acid with the available calcium of the substrate, and a micro-mechanical interlocking of the resin matrix. It is, however, still unclear to what extend each of these two bonding mechanisms contribute to the actual adhesion [26]. Thus, when RMGI is chosen, it is recommended that dentin should be lightly roughened and prepared with a rotary instrument in order to remove the outer surface layer of the sclerotic dentin. The use of inverted cone burs is preferred because of their short length, to reduce the risk of injuring gingiva during use of the bur in subgingival class V preparations [7]. The smear layer interference that is created from this process must be excluded with the use of a polyalkenoic-acid conditioner, enabling the RMGI to interact with the tooth substrate [26]. The conditioner should be applied and removed with attention so the dentin is not over-dried [6]. Although conditioning is unnecessary when the sclerotic dentin is removed with laser irradiation, this alternative method of preparation should be avoided because RMGI interacts very superficially with laser-irradiated dentin, adversely influencing the bonding efficacy of RMGI [4,26]. Besides the elimination of the smear layer, some studies suggest that enamel should be edge beveled in order to allow the glass ionomer to have adequate thickness at the margins, which compensate for its low fracture strength [7,10]. When conditions, such as those in hospital patients, do not allow the application of burring, the use of conventional glass ionomer may be a better option. Shear bond strength of conventional GIC to sound dentine is shown to be similar to that of sclerotic dentin [11]. In that case, the absence of a smear layer makes the use of cavity conditioner unnecessary [26]. In any case, before the placement of the restorative material it is recommended that cavity surfaces be cleaned, especially when the ART technique is used. For that purpose, the use of a pumice-water paste or 2% solution of chlorhexidine-gluconate is suggested [7,27,28], while the use of sodium hypochlorite or an 810 nm diode laser and a photo-sensitizer, which has been proposed, should be avoided [28].

As soon as the preparation of the lesion is completed, the clinician is ready for the placement of the restorative material. When RMGI is chosen, the product specific cleanser/primer is used [7]. After this step, it is time for the cement to be placed. The material can be applied either with an auto-mixing capsule after it is activated or by hand carving [6]. RMGI should be built in increments no greater than 3 mm in depth so that it can take advantage of the improved physical properties that can be developed through irradiation [29]. Hand-carved conventional glass ionomers, on the other hand, should not be touched for several seconds and then edited with the minimum number of strokes needed, moving from the center of the material to each side for a total of at most three to five strokes [6]. In both cases, a cervical matrix should be applied before the curing of the material. Cervical matrices vary in shapes and sizes for anterior teeth, premolars and molars, and clinically allow the material to be adapted to the margins of the preparation under pressure, providing for a leak-free restoration [7]. After the adaptation of the cervical matrix, the RMGI restoration should be light cured for the manufacturer’s recommended time, which is usually 20 s with a high-intensity LED light curing unit [7].

Both conventional and resin-modified glass ionomers require a coating. This could be a varnish or a light-activated resin enamel bond. Conventional glass ionomers need to be sealed as soon as possible after setting, to protect from immediate moisture. Resin-modified glass ionomers can be finished and polished immediately, and the coating is recommended to prevent from moisture over the next seven days. Conventional glass ionomers should be finally polished after at least 24 h, in a separate appointment. The sealing process may then need to be repeated [6].

Overall, the question is which glass ionomer cement a clinician should choose for the restoration of NCCL in geriatric patients, the conventional or the resin modified. Improved mechanical properties, quicker set reaction and better aesthetics are the characteristics of RMGI [11,29], which make it, generally, the restorative material of choice. However, in some cases, when the general and oral health of frail and functionally dependent older patients creates limitations, the use of conventional GIC should be preferred. Conventional glass ionomers are the optimal choice for treatment of xerostomic patients owing to radiation, medication, salivary gland disorders, etc., and for generally high caries risk patients. Although RMGI appear to have better resistance against abrasion and erosion, this is not the case in xerostomic mouths. The purpose of restoration here is the protection from secondary caries. Active caries are observable three months after radiotherapy and extreme damage of the dentition is commonly seen within a year. The presence of caries in these patients progresses rapidly and can lead to full fracture of the crown [11,30]. Conventional glass ionomers release fluoride to a higher extent than RMGI [12,15] and therefore provide better protection against secondary caries. If this material fails due to erosion and dehydration of the dry mouth, the failing restorative materials may be replaced [30]. In these cases coating the surface of the restorative material, to prevent contamination with moisture during the initial stages, is to be avoided, because it results in a decrease of fluoride release up to 1.4–4 fold [15]. However, attention is needed when the patient uses oral gel or a solution of fluoride. Topical fluoride formulations, such as acidulated phosphate fluoride (APF) gel, can damage the surface layer of the restoration, and so it is necessary for the restorative surface to be sealed. In contrast, neutral sodium fluoride 2% gel does not affect the surface of the cement, and no coating is needed [11].

**Figure 1:**
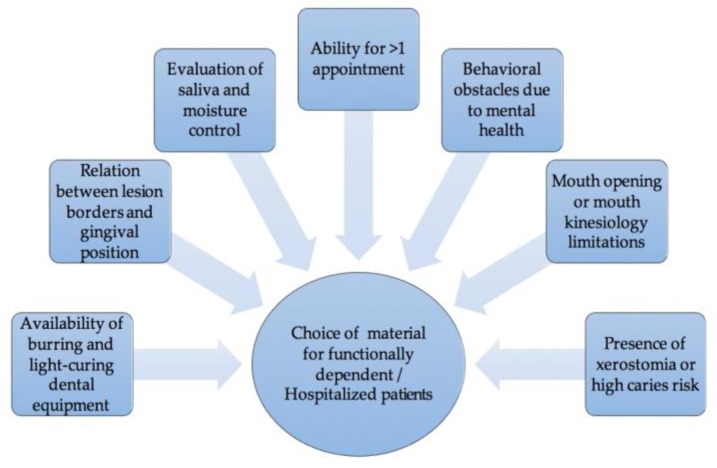
Factors to be considered when choosing a material for functionally dependent/hospitalized patients.

**Figure 2:**
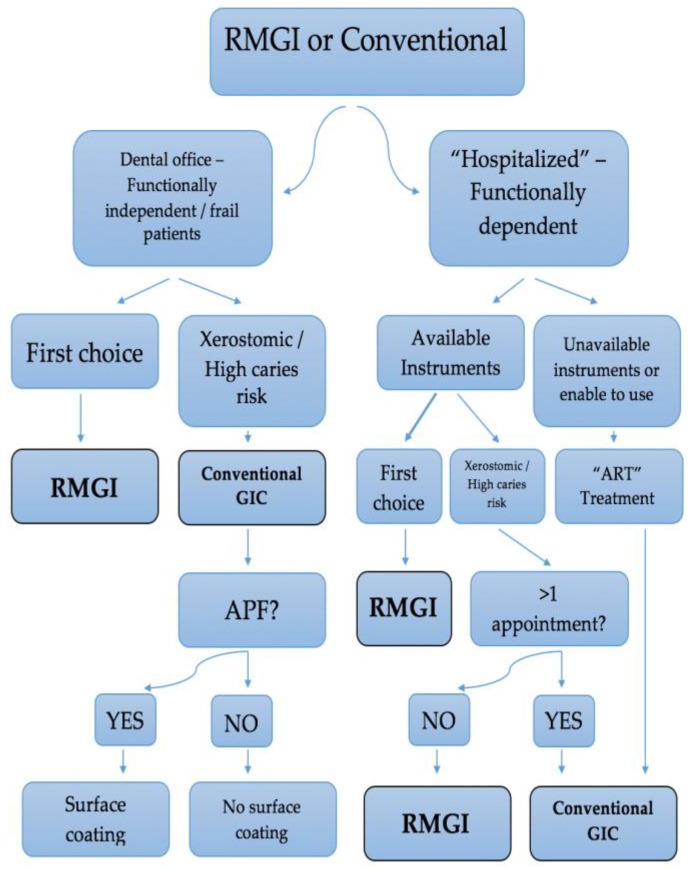
The process of choosing a restorative material for non-carious cervical lesions (NCCL) in geriatric patients.

When it comes to hospitalized geriatric patients or patients who are not capable of visiting a dental office, a clinician should include several factors to pick the most suitable material (Figure 1). The availability of burring and light curing dental equipment, the relation of the position of the lesion and the gingiva, the evaluation of saliva and moisture control, the lack of opportunity for more than one appointment, the presence of xerostomia, health conditions which affect mouth opening or mouth kinesiology, and behavioral obstacles are all issues to be considered. Ideally, when a portable dental office can be used, RMGI should be chosen, just like at a dental office. In xerostomic patients and in subgingival wet environments, conventional glass ionomers are again the first material choice, except if a second appointment for the finishing and polishing of the restoration is impossible. Otherwise, when the essential dental equipment cannot be transported, or the oral and mental condition of the patient do not allow it to be used, the Atraumatic Restorative Treatment should be applied. Features, such as bonding to sclerotic dentin [11], maintenance of adhesion for long periods and high retention rates [5], high fluoride release [15] and compatibility with the thermal expansion coefficients of hard tooth tissues, that make mechanical retentions unnecessary and save the remaining tooth structure [5] make conventional glass ionomers the material of choice for the ART approach [14]. It is important to remember that before the placement of the restoration the lesion should be disinfected with use of a pumice-water paste or 2% solution of chlorhexidine-gluconate, in order to decrease the microbial load, as mentioned above. The process of choosing the restorative material for NCCL in geriatric patients is summarized in Figure 2.

## 8. Conclusions

Glass ionomer cement is considered to be the material of choice for the restoration of NCCLs in geriatric patients. There is no ‘typical’ older person, therefore the restorative management of these lesions in older adults should be adjusted accordingly to the patient’s profile. In general, RMGICs appear to be preferred, but under certain circumstances the use of the conventional product is more appropriate, despite its poorer mechanical features. However, the evidence for these guidelines is limited, and further studies should take place in this direction.
